# Mechanisms of Post-critical Illness Cardiovascular Disease

**DOI:** 10.3389/fcvm.2022.854421

**Published:** 2022-07-15

**Authors:** Andrew Owen, Jaimin M. Patel, Dhruv Parekh, Mansoor N. Bangash

**Affiliations:** ^1^Department of Critical Care, Queen Elizabeth Hospital, University Hospitals Birmingham, Birmingham, United Kingdom; ^2^Birmingham Acute Care Research Group, Institute of Inflammation and Ageing, University of Birmingham, Birmingham, United Kingdom

**Keywords:** critical illness, chronicity, atherosclerosis, insulin resistance, inflammation, heart failure, immune aging, CKD

## Abstract

Prolonged critical care stays commonly follow trauma, severe burn injury, sepsis, ARDS, and complications of major surgery. Although patients leave critical care following homeostatic recovery, significant additional diseases affect these patients during and beyond the convalescent phase. New cardiovascular and renal disease is commonly seen and roughly one third of all deaths in the year following discharge from critical care may come from this cluster of diseases. During prolonged critical care stays, the immunometabolic, inflammatory and neurohumoral response to severe illness in conjunction with resuscitative treatments primes the immune system and parenchymal tissues to develop a long-lived pro-inflammatory and immunosenescent state. This state is perpetuated by persistent Toll-like receptor signaling, free radical mediated isolevuglandin protein adduct formation and presentation by antigen presenting cells, abnormal circulating HDL and LDL isoforms, redox and metabolite mediated epigenetic reprogramming of the innate immune arm (trained immunity), and the development of immunosenescence through T-cell exhaustion/anergy through epigenetic modification of the T-cell genome. Under this state, tissue remodeling in the vascular, cardiac, and renal parenchymal beds occurs through the activation of pro-fibrotic cellular signaling pathways, causing vascular dysfunction and atherosclerosis, adverse cardiac remodeling and dysfunction, and proteinuria and accelerated chronic kidney disease.

## Introduction

The global burden of critical illness is difficult to quantify due to many factors but even prior to the Coronavirus Disease 2019 (COVID-19) pandemic it was likely to be substantial and broad reaching (1). Traditionally the focus of large multi-center randomized controlled trials in critical illness has been on interventions aimed at decreasing inpatient mortality, but the spotlight is increasingly turning to post-critical illness survivorship and quality of life (2–4). Several recent studies highlight that even in previously healthy individuals a period of critical illness is associated with the onset of long-term chronic health problems. In this review we critically appraise the literature surrounding post critical illness cardiovascular disease and explore the potential mechanisms.

## Surviving Critical Illness

Admission to critical care usually marks a patient's most severe lifetime episode of acute illness. Two fundamental lessons can be learned from critical illness survivor studies—the effects of critical illness are not short-lived or trivial, and the severity of these long-term effects relate to the severity and duration of the acute illness. For example, 3-year mortality in survivors of critical illness is elevated compared to non-critically ill patients, and these elevations in mortality are considerably increased by higher critical illness severity (5). Similar associations between illness severity, duration and one-year mortality are also found in ARDS when examining survivor data (6–8). Although co-morbidity and advanced age deleteriously synergise with acute illness severity, studies attempting to correct for this still show an elevated subsequent mortality associated with sepsis and acute respiratory failure diagnoses (8–11). The recent COVID-19 pandemic has served to emphasize these matters though intensive care unit (ICU) follow-up has naturally been shorter in this disease than for other critical illnesses (12).

Examination of the temporal patterns of mortality in ICU survivors (compared to hospital controls) shows a rapid cumulative excess mortality over the first 6-months following discharge, which slows in its accrual rate over the next 2.5 years of follow-up (5). One study found that over 35% of ICU survivor deaths within 1-year of ICU admission relate to cardiovascular, renal and cerebrovascular diseases and interestingly this cluster of diseases was ahead of malignancy as the leading cause of post-ICU survivor deaths (13). Other studies also point to elevated rates of cardiovascular and renal diseases and associate them with blood-stream markers of inflammation, showing an accelerated mortality which gradually settles with time (8, 14).

The question arises as to whether this is a generalizable phenomenon or whether there are only specific subsets of the critically ill to whom this applies. Data from previously young healthy adults who have survived severe traumatic injuries show an increased rate of new onset cardiovascular disease (15). Furthermore, there is a dose-response relationship between severity of injury (by injury severity score) and cumulative incidences of hypertension, coronary artery disease, diabetes mellitus, and chronic kidney disease (CKD). These very same diseases are also seen in adult burn patients (16, 17). Trauma and burns, as with many other critical illnesses, are often associated with acute kidney injury (AKI). Data from a retrospective study in Denmark demonstrated a one-year mortality rate of over 50% following dialysis-requiring AKI (though not strictly isolated to the critically ill), and a 1-year end-stage renal failure rate of over 5% (18). Similarly, follow-up of a large randomized controlled trial of differing dialysis intensities for AKI in critical care also showed that the overall 1-year mortality rate following AKI is over 50% with the majority of deaths occurring in the first 90 days, but with a significant and ongoing mortality over several years (19). The aforementioned study found an association between the severity of critical illness (determined by APACHE III score) and the risk of long-term mortality and also showed the association of AKI survival with high rates of cardiovascular disease, proteinuria and CKD. There are obvious parallels with the findings of studies in trauma and burns where injury severity correlated with subsequent cardiovascular morbidity (15). Sepsis studies show similar trends of elevated cardiovascular risk in survivors, as do studies of viral [e.g., influenza and severe acute respiratory syndrome coronavirus 2 (SARS-CoV-2)] and bacterial respiratory infection (20–26). Interestingly, the elevated cardiovascular risk seen in sepsis survivors is similar to non-infected critically ill controls in some studies, suggesting that this phenomenon is either not infection specific, or that non-infection diagnostic categories fail to adequately capture same-stay inpatient infection/sepsis events (20).

Were this phenomenon universal, one would also expect it to be recapitulated in the pediatric setting. Pediatric critical care survivor data suffers from problems of a relative sparsity coupled with inclusion of large numbers of patients with confounding inherited or congenital co-morbidities. Furthermore, pediatric survivorship is usually coupled with continued physical growth and maturation which has the potential to offset pathological adversity. Nevertheless, there are data that supports a generalisability of the phenomenon seen in critically ill adults. Children who have suffered AKI (whether in ICU or not) show a similar pattern of progression to proteinuria, CKD and end-stage renal disease later in adulthood (27, 28). In the pediatric burn population, studies show increased rates of type 2 diabetes mellitus (T2DM), cardiovascular disease and significant myocardial and circulatory impairments in survivors (17, 29, 30). Irrespective of age, surviving a wide variety of severe critical illnesses results in an accelerated onset of chronic disease, particularly that which relates to the cardiovascular and renal systems.

## Cardiovascular Disease—The Traditional Model

The constellation of cardiovascular diseases comprising hyperlipidaemia, essential hypertension, coronary heart disease, cerebrovascular disease, heart failure, and CKD have substantial clinical overlap (31–35). Although it was over a century ago that Virchow suggested involvement of the immune system in atherosclerosis based on post-mortem studies, the understanding of the pathogenesis of atherosclerosis came to center on abnormalities of lipid homeostasis (36). Hypertension research only became possible after the introduction of routine and non-invasive blood pressure measurement, and associations between hypertension and cardiovascular diseases such as atherosclerosis, heart failure, or stroke soon became apparent (37). Abnormalities of salt and water homeostasis and autonomic regulation as drivers of hypertension were considered important, implicating renal, humoral, and neural mechanisms alongside abnormalities in lipid metabolism as drivers of cardiovascular disease. Other common findings that were considered important but assumed to have secondary roles in the pathophysiology of cardiovascular disease related to the immune infiltrates that Virchow and others had observed, subsequent oxidative and free-radical mediated damage, microvascular dysfunction with blunted endothelial-dependent vasodilatory responses, extracellular matrix remodeling and fibrosis, and platelet activation. Many of these secondary findings are also seen in T2DM, though the underlying driver there is believed to be pathologically elevated plasma glucose levels and tissue insulin resistance.

## Chronic Inflammation and Cardiovascular Disease

Although inflammation has long been recognized as playing a potential causative role in cardiovascular disease (particularly atherosclerosis), significant doubt existed whether detected inflammation was a result or cause of atherosclerotic disease (38, 39). Although acute inflammation can be shown to associate with elevated cardiovascular risk in humans, definitive proof that inflammation is an independent mechanistic driver of human cardiovascular disease was until recently lacking. Clinical studies suggest that modifying inflammation may hold cardiovascular benefit (40, 41), while some animal studies have also confirmed accelerated cardiovascular pathology following inflammatory insults (42, 43). It is only recently that the role of inflammation as a therapeutic target for combating cardiovascular disease has been confirmed by the CANTOS trial, where interleukin (IL)-1β antagonism showed a reduction in composite cardiovascular outcomes in patients with established coronary artery disease independent of plasma low density lipoprotein (LDL) cholesterol (44–46). While this is an exciting development, the mortality benefits of therapy in this setting overrode, but nonetheless, were accompanied by an increased likelihood of death due to infection, a particularly important finding for survivors of sepsis and critical illnesses who have a high rate of infection-related hospital & ICU readmissions (47).

## Post-Critical Illness Morbidity and Mortality

### The Critical Illness Paradox

Given that inflammation is an aetiological factor in chronic cardiovascular disease, we hypothesize that critical illness is a phase of hyperinflammation that can drive *de novo* or accelerate pre-existing cardiovascular morbidity and mortality. Critical illness is undoubtedly associated with inflammation and survivor chronic illness and mortality, particularly of cardiovascular origin (14). However, it is not immediately obvious how relatively brief acute illnesses that have lasted only days to weeks and that end in a repair and resolution phase of illness can induce a step change in cardiovascular risk. In this phase the levels of many pro-inflammatory cytokines have decreased, patients are often normotensive (and may have spent more time during their illnesses with lower than normal blood pressures), with low levels of total cholesterol, and may have low circulating levels of cortisol and aldosterone. Patients also display functional immunosuppression (48), and so this constellation of features would appear to run counter to the traditional and inflammatory models of cardiovascular disease (see Table 1). However, by turning to modern insights in to the pathogenesis of cardiovascular disease this apparent paradox is somewhat resolved.

**Table 1:** The paradox of a lack of traditional risk factors for renal and cardiovascular disease during critical illness.

**Risk factor**	**Hypertension**	**Insulin** **resistance**	**Elevated** **cholesterol &** **TG**	**Elevated cortisol**	**Elevated** **aldosterone** **levels**	**Immune response**	**Age**
Chronic CVS/renal disease	Recognized risk factor	Recognized	Recognized	Associated with HT, DM, & insulin resistance	Recognized	T-cell, monocyte, & macrophage activation recognized	Recognized
Critical illness	Not a feature of patients with prolonged shock	Common during acute illness	Usually less than baseline and often in low/normal range	Plasma levels inappropriately depressed in many	Plasma aldosterone may be depressed	T-cell anergy, myeloid derived suppressor cells found	Trauma/burns tends to affect young, sepsis affects all groups (elderly and pediatric groups more so)

*Although there is a documented high rate of cardiovascular and renal morbidity in the months to years following critical illness, many of the risk factors associated with these diseases are not obviously seen in critical illness*.

### Critical Illness Related Oxidative Damage Triggers “Chronic Disease Like” Immune Activation and Endothelial Dysfunction

Considering that plasma endotoxin levels associate with incident diabetes and cardiovascular disease in the community setting, there is at least at the epidemiological level a strong association between innate immune activation and the development of metabolic and cardiovascular diseases (49–51). One mechanism of this linkage is suggested to be through molecular pattern receptor activation triggering low grade inflammation and cytokine mediated insulin resistance (52). There is no reason to suspect such phenomena are isolated to chronic disease and do not occur alongside or as a consequence of acute inflammatory events such as those which cause critical illness:

The acute phase of critical illness is undoubtedly characterized by tremendous immune activation and consequential free radical mediated damage (53, 54). Myeloperoxidase (MPO) is an important enzyme in the process of oxidative damage, it is the most abundant enzyme found in neutrophils, and is capable of inducing microvascular dysfunction by decreasing endothelial nitric oxide bioavailability, both independently and when complexed with high density lipoprotein (HDL) (55, 56). Furthermore, under various conditions, including pre-clinical models of sepsis, the free-radical mediated damage by MPO and other enzymes such as NADPH Oxidase (NOX) results in the formation of highly reactive isolevuglandins (γ-ketoaldehydes formed from the isoprostane pathway) that rapidly modify cellular proteins and activate pro-inflammatory pathways in tissue antigen presenting cells (57–60). Isolevuglandin protein adducts, found at elevated levels in patients with atherosclerosis and end-stage renal disease, are presented by dendritic cells bringing about monocyte recruitment to the endothelium, linking free-radical mediated damage with the activation and further recruitment of immune cells to the vasculature (61). Furthermore, these protein adducts modify LDL resulting in the uptake of LDL by receptors for oxidized LDL on macrophages, and they modify HDL, resulting, resulting in dysfunctional circulating HDL (62). Although leucocyte-endothelial interactions necessarily increase at sites of infection or tissue injury through mechanisms that are independent of this specific pathway, isoprostanes have also been detected at elevated levels in patients with sepsis (63). This suggests that this pathway, which is of importance in hypertension and atherosclerosis, could be of importance in human post critical illness cardiovascular pathogenesis.

HDL is considered anti-inflammatory, atheroprotective, and inhibitory of microvascular dysfunction by virtue of its myriad effects on inflammatory signaling and adhesion molecule upregulation, foam cell cholesterol efflux, and inhibitory effects on LDL oxidation and Toll-like receptors (TLRs) (64). It serves as a carrier molecule for transferring endotoxins such as lipopolysaccharide (LPS) and lipoteichoic acid (LTA) from the bloodstream to the liver where they can be rapidly cleared and detoxified by enzymes such as acyloxyacyl hydrolase, but HDL also carries other molecules such as the enzyme symmetric dimethyl arginase (SDMA) which limits nitric oxide bioavailability in the circulation (65, 66). It has been shown in some patient groups that share clinical overlap with critical illness survivors (e.g., those with CKD and T2DM) that HDL is abnormally modified by isoprostanes as described above, or by the presence of SDMA, in both cases inducing endothelial dysfunction and atherosclerosis *via* the activation of macrophage TLRs and inhibition of nitric oxide (67–69). One study showed fifty percent of 28-day sepsis survivors have SDMA levels of 0.82 μmol.l^−1^ or more, which is a level where population nomograms show severe HDL impairment (70, 71). The amount of circulating functional HDL is therefore also likely low in a substantial number of sepsis, and possibly other previously hyperinflamed, ICU survivors. This is not to implicate these pathways as being central drivers of the severe microvascular dysfunction which occurs during critical illnesses such as sepsis (though it may partly explain why patients with pre-existing cardiovascular diseases have worse basal microvascular function than others). Instead we propose that these pathways become relevant for ongoing vascular dysfunction after the critical phase of illnesses have subsided.

Microvascular dysfunction has been found in a number of different types of critical illnesses and is associated with endothelial damage and disruption of the endothelial glycocalyx (72). Due to the inability to measure basal microvascular health before admission (except in elective surgical patients), no studies have specifically looked at the rate of normalization of microvascular abnormalities in individuals after critical illnesses, which makes it difficult to assess the potential importance of these pathways in convalescence. However, one study in sepsis assessed biomarkers of endothelial health from ICU admission to 1-year out. This study showed that in 14–38% of surviving patients, levels of all measured soluble endothelial adhesion molecules and markers of coagulation activation [e.g., plasminogen activator inhibitor (PAI) −1] remained elevated above normal from 90 days after admission until a year out (73). Many of these patients have persistently elevated levels of IL-6, inducing both the endothelial release of PAI-1 and the upregulation of endothelial adhesion molecules, suggesting that the ongoing presence of inflammation and circulating cytokines such as IL-6 sustains endothelial activation (74–76). Furthermore microalbuminuria, a condition that is highly associated with systemic microvascular dysfunction, is particularly common after admission to ICU in those with severe kidney injuries (19, 77). Therefore, abnormalities in endothelial health and microvascular function probably persist after severe critical illnesses for a significant period of time in a substantial proportion of ICU survivors. As endothelial dysfunction is also involved in the initiation and progression of atherosclerotic disease its persistence is likely to contribute to ongoing cardiovascular disease. This paradigm of a seamless progression of endothelial dysfunction from one disease state to another is also supported by conserved inflammatory signaling pathways that induce endothelial activation both in peritonitis and atherosclerosis (78, 79). Furthermore, recent findings that chronic microvascular rarefaction precedes tissue aging suggests that critical illness could induce a step-change toward chronic organ dysfunction and tissue aging at least through the premature induction of chronic vascular dysfunction (80).

### Acute Myocardial Injury, Dysfunction and Its Aftermath

The fourth universal definition of myocardial infarction provides a framework for understanding increases in the cardiac injury biomarker Troponin (cTn), —essentially dichotomising to acute myocardial infarction of a variety of ischaemic subtypes and causes external to it (non-ischaemic injuries) (81). Although non-ischaemic myocardial injuries do not involve clinically detectable ischaemia, they can be contributed to by processes that can cause ischaemic injuries and myocardial infarction (e.g., anemia, sustained and severe tachycardia); correctly differentiating non-ischaemic from ischaemic injuries is therefore difficult but improved by rigorous clinical ischaemia assessment (82). Increases in cardiac injury biomarkers such as cTn are common during critical illnesses and are not always accompanied by overt myocardial ischaemia, dysfunction or shock. However, myocardial injury in critical illness is independently associated with adverse acute disease outcomes (83, 84). Outside of ischaemia organ-specific extra-cardiac processes such as pulmonary embolism, hyperadrenergic-associated myocardial injuries in a variety of brain injuries, and polytrauma with myocardial contusion can contribute to cTn release (85–87). Notwithstanding that, there are substantial numbers of critically ill patients who see increases in cTn without fitting in to the aforementioned etiologies. These patients may appear completely stable, such as the routinely observed post-operative major surgical patient with a peri-operative myocardial injury, or alternatively may have severe shock and multiple-organ failure due to a variety of potential causes.

Elevated cTn levels in this context are not dependent on a prior history of ischaemic heart disease or congestive heart failure, though levels tend to be higher in those with such a history (88). Similarly while renal impairment can somewhat affect the circulating levels of cTn through decreased elimination, it is erroneous to dismiss new or large increases in cTn to renal dysfunction alone—cTn clearance is less reliant on renal clearance as blood concentrations increase following its release in to the circulation (89, 90). Furthermore, the persistently raised cTn that is commonly seen in CKD outside of critical illness is also likely to be minimally contributed to by renal impairment *per se*, implying ongoing myocardial injury instead (91). However, the presence of shock, commonly associated with myocardial dysfunction and usually requiring vasoactive/inotropic drugs, is associated with a higher degree of cTn release than non-shocked states (88, 92). Similarly higher degrees of ARDS severity associate with higher levels of cTn, and also associate with worse endothelial function (93). Other observational studies in mixed cohorts of critically ill suggest associations of cTn with IL-6 and inflammatory state, which is further supported by peri-operative studies (94, 95). Sympathoadrenal hyperactivation in the context of inflammation and tissue injury, proposed to induce shock-induced-endotheliopathy (SHINE), is a potentially contributory mechanism to cTn release, though clearly sympathetic activation is not unique to shock and occurs to a greater or lesser degree in all critically ill patients so that it could partially account for cTn elevation outside of shock states too (96–98). Our findings of an independent association of both heart rate and markers of vasoplegia with hyperdynamic left ventricular function (where cTn release is higher than any other echocardiographic phenotype of septic shock) lends further support to the SHINE concept (99).

In line with this angiography studies in patients with post-operative ACS demonstrate little in the way of obstructive coronary artery disease or plaque thrombosis, but instead show a high prevalence of Takotsubo's cardiomyopathy, a catecholamine-mediated condition (100, 101). Similarly a study in critically ill medical patients also showed Takotsubo-type left ventricular apical ballooning in approximately one third of patients (102). Critical illness related myocardial injury may therefore occur through a combination of sympathoadrenal and cytokine induced cardiomyocyte dysfunction and death—these can drive immunogenic and destructive regulated cell death processes within the tissue microenvironment, such as necroptosis and pyroptosis (103, 104). These latter processes are preceded by and result in the further release of large amounts IL-1β and IL-6. Although some histological studies suggest cell death is infrequent in septic myocardial injury, cardiomyocyte death can be driven by very brief ischaemia that does not produce overt infarction (105). This can drive inflammatory death in macrophages in the cardiac microenvironment through cytosolic nucleic acid detection and the triggering of type 1 interferon responses, implying a self-reinforcing loop of cardiomyocyte death and inflammation (106). However, sources of nucleic acids beyond those in necrosing cardiomyocytes also exist, such as circulating mitochondrial and cell free DNA in trauma, and cytosolic DNA due to sub-lethal cell stress (107, 108). The cardiovascular dysfunction that features as part of shock in critical illness is associated with leukocyte infiltration of the myocardium, myocardial oedema, electrophysiological and cardiomyocyte contractile dysfunction (109). These abnormalities are thought to have their origins in enhanced adrenergic signaling with increased oxidative and nitrosative cell stress, abnormalities of mitochondrial structure and function, abnormalities of calcium sparking, and also possibly through alteration of cardiac macrophage behaviors which are normally involved in co-ordinate action potential propagation through Connexin-43 mediated interactions with cardiomyocytes (109–111). Pre-clinical studies have shown disruption of cardiac gap junctions and Connexin-43 in both septic and traumatic cardiac dysfunction, suggesting this may be involved in arrhythmogenesis, such as atrial fibrillation (AF) (112, 113). The finding that β-adrenoceptor provoked S-nitrosylation of Connexin-43 is arrhythmogenic in pre-clinical models of inherited cardiomyopathies is also notable in this regard (114).

New-onset AF occurs in roughly one in five critically ill patients, predominantly in a paroxysmal fashion, and is associated with the risk of in-hospital death (115, 116). It occurs sub-clinically in a high proportion of patients and therefore may affect patients without being detected. One follow-up study of critical illness survivors demonstrated an association of ICU-acquired AF with 1-year post-discharge mortality, while the MANAGE trial which investigated the pharmacological management of peri-operative elevated cTn in those having major non-cardiac surgery (a population which is highly prevalent in surgical ICUs) showed improved outcomes in patients randomized to the anti-coagulant dabigatran (116, 117). In a pre-planned secondary analysis of MANAGE the reduction in the composite primary outcome (a composite of vascular mortality and non-fatal myocardial infarction, non-haemorrhagic stroke, peripheral arterial thrombosis, amputation, and symptomatic venous thromboembolism) was seen to be significantly reduced only for non-haemorrhagic stroke, a diagnostic category with strong aetiological association with AF. Furthermore, when looking to survivors of sepsis, those with new onset AF during their acute illness were confirmed in another study to have higher risks of post-critical illness mortality, stroke and also recurrent AF and heart failure than those without (118). New onset AF may then associate with AF in convalescence, the importance of this being that AF is a risk factor for both stroke and heart failure. Given a link between (post-operative) IL-1β release, abnormal cardiomyocyte calcium handling and AF has already been established, it may be that residual inflammation and metabolic dysfunction are targetable drivers of post-ICU AF (119, 120).

Prolonged exposure of the injured myocardium to catecholamines is associated with fibrotic change (121). Studies in patients with Takotsubo's cardiomyopathy show (iron-laden) monocyte/macrophage infiltrates in the myocardium which would seem to be akin to monocytic infiltrates seen in sepsis (122). Pre-clinical studies also support the role of iron-processing macrophages in septic cardiac dysfunction through the enhancement of nitric oxide production (which leads to a vicious cycle of increased catecholamine dosing through reduced adrenoceptor sensitivity), and is also in keeping with studies in human heart failure (123–126). Although Takotsubo studies show echocardiography normalizes some months after the initial event just as echocardiographic abnormalities normalize soon after sepsis, there is still associated residual poor functional ability, ongoing systemic inflammation, magnetic resonance imaging evidence of diastolic dysfunction and a persistently abnormal energetic status common to heart failure (122, 127). Remarkably heart failure, particularly that with preserved ejection fraction (HFpEF), commonly occurs after critical illness in groups without a prior cardiac history (e.g., after severe pediatric burn) suggesting that the acute intracardiac nitrosative stress that is involved in acute cardiac dysfunction remains latent and plays an important aetiological role in HFpEF pathogenesis (29, 126). It is also notable that the recently devised HFpEF score marks elevated body mass index (BMI) and AF as high scoring risk factors for the likelihood of HFpEF—both of these risk factors are prevalent in survivors of critical illness (128). Thus, patients surviving severe critical illnesses with myocardial injury and prolonged periods of high catecholamine exposure (not necessarily exogenous) are likely to develop myocardial inflammatory infiltrates which in the context of ongoing inflammation, neurohumoral activation [including components of the renin-angiotensin-aldosterone system (RAAS)] and ongoing preferential adipose deposition, could develop in to latent heart failure through immune-mediated mechanisms that stimulate myofibroblast proliferation (124).

### Cardiorenal Synergy in Chronic Cardiovascular Pathogenesis

The immune response to AKI is associated with increased levels of pro-inflammatory cytokines and immune cell secreted Galectin-3, the latter being implicated in myocardial inflammation and fibrosis in acute and chronic settings (129, 130). Sympathoadrenal and neurohumoral activation occur in the acute phase of severe critical illness, as exemplified by sepsis (131, 132). Galectin-3 expression and secretion is increased in response to the terminal RAAS mineralocorticoid hormone aldosterone, salt intake, and beta-adrenoceptor agonism (133–135). However, these sympathoadrenal and neurohumoral changes are also important in driving chronic disease through oxidative damage. Renal nerve derived noradrenaline and circulating Angiotensin II [Ang II; tightly linked to NOX *via* the type 1 angiotensin II (AT_1_) receptor] are recognized vasoconstrictive mediators of chronic hypertension, and have also been shown to induce the formation of renal dendritic cell isolevuglandin protein adducts (136). Antigen presentation by these dendritic cells activates and drives renal T-cell infiltration with attendant renal pro-inflammatory cytokine release and altered renal histology (136, 137). AKI studies also show the macrophage chemokine CCL-14 as a highly predictive biomarker of persistent (>72 h) high grade AKI (138). As higher grade AKI and acute kidney disease (AKD; AKI of 7 days or more duration) is predictive of CKD progression this suggests renal macrophages are involved in imperfect repair processes that cause renal fibrosis (139). This is consistent with data from animal studies of AKI showing that renally secreted transforming growth factor (TGF)-β and damage associated molecular patterns (DAMPs) draw in macrophage and T-cell populations which drive renal fibrotic repair programs as part of a cycle of ongoing tubular cell death and macrophage recruitment and activation (140, 141). One study showed that renal mesenchymal cells such as fibroblasts and pericytes are the main cell population that become activated myofibroblasts, driving extracellular matrix deposition and renal fibrosis in response to TGF-β and platelet-derived growth factor (PDGF) signaling (142). Although this study didn't implicate immune cell infiltration as a major component of CKD progression, the model proposed is nonetheless in keeping with one which involves parenchymal and macrophage derived PDGF and TGF-β as initiators of chronic fibrosis in the early stages of injury and is also similar to fibrotic circuits found in the heart (124, 143). As Galectin-3 stabilizes cell surface cytokine receptors and inhibits T-cell apoptosis it may hinder inflammation resolution across the cardiovascular and renal systems, thus skewing toward maladaptive repair and chronicity following AKI (144).

Importantly, although AKI is likely a multi-faceted process and varies in its pathophysiology depending upon the nature of the initiating injury (e.g., sepsis vs. polytrauma), the transition to fibrotic repair and CKD is transcriptionally preserved among different types of CKD pathophysiology and implicates the immune system, renal parenchymal hypoxia, and pro-fibrotic growth factor signaling molecules such as TGF-β (145–147). The early understanding of idiopathic hypertension as being secondary to changes in the kidneys may therefore partly hold true for post critical-illness induced hypertension, potentially aligns renal microvascular dysfunction and microalbuminuria, and might also explain the beneficial effects on mortality of post-AKI Angiotensin Converting Enzyme inhibitors (ACEi) in observational studies (77, 148, 149). The interaction of vascular, renal and heart diseases as is currently understood for chronic disease therefore also apply in the setting of acute illness and its repair phase, though the specifics of the interaction after critical illnesses are unique to acute injury and repair programs, probably occur at a more rapid pace than occur naturally in aging, but clearly only become a concern for patients that can survive the resuscitative phases of illness (150, 151). Furthermore, the persistence of CKD progression may contribute, alongside inflammatory programs, to sarcopenia and frailty in convalescence (152).

### The Immune Response to Tissue Injury Leads to Altered Systemic Metabolism and Adiposity Which Exacerbates Cardiovascular Homeostasis

Amongst the most obvious metabolic changes that occur in critical illnesses are early increases in circulating levels of lactate and glucose, but there are other changes which are just as obvious but perhaps less appreciated. The onset of illness leads to sickness behavior favoring the diversion of energy utilization—a reduction in physical activity is partly possible due to a cytokine mediated induction of lethargy and fatigue and also a reduction in appetite that neutralizes the urge to seek food. Changes in appetite are partly regulated by the IL-1β induced hormone growth differentiation factor (GDF)-15 which signals *via* central nervous system glial cell derived neurotrophic factor (GDNF) family receptors α-like (GFRAL) receptors to induce anorexia (153). The energy furnished by sickness behavior and tissue breakdown is diverted toward powering the febrile response, increased cardiac work, and the systemic response required for tissue repair and immunity. For example, pre-clinical studies suggest that the GDF-15-GFRAL signaling pathway induces centrally driven sympathetic output to the liver which supports hepatic triglyceride output. This may provide an energy substrate for the heart and other tissues despite an IL-1β mediated downregulation of VLDL receptors in the septic heart, thus enhancing tolerance of cardiac injury in the face of sepsis (154–156). Notably, GDF-15 signaling induces muscle breakdown in studies inside and outside of ICU, thus contributing another systemically available fuel source in illness (157–159). Furthermore, the hormone is also seen as a biomarker of mitochondrial health and cellular stress and senescence as it increases with increasing cellular and organismal dysfunction (160). Whether the linkage between high GDF-15 levels and poor ICU outcomes is related to its contribution to cachexia remains unknown, but levels of this hormone remain elevated in ICU survivors for at least 1 week after ICU discharge so that potentially undesirable effects of GDF-15 could persist in convalescence (161).

Concurrent decreased levels of LDL cholesterol and increased levels of triglycerides and free fatty acids (FFAs) have been observed in critically ill patients, and may occur partly due to increased hepatic triglyceride output, early catecholamine driven increases in adipose tissue lipoprotein lipase and hormone sensitive lipase activity, and additional factors that take precedence at different times such as dynamic reductions in non-adipose lipoprotein lipase activity (162–164). Both obesity and critical illness see high levels of macrophage and other immune cell infiltrates in adipose tissue which contribute to adipose-related inflammation and insulin resistance in the outpatient setting; the role of these immune infiltrates beyond possibly serving as immune memory is unknown in critical illness convalescence (162, 165). However, the serine-phosphorylation of insulin-receptor-substrate-1 by cytokines and TLR4 agonists such as FFAs means that convalescent obesity and adverse convalescence metabolic profiles in addition to residual inflammation can be expected to perpetuate insulin resistance (166, 167). As the normal action of insulin is to facilitate glucose uptake *via* glucose transporter type 4 (GLUT4) channel expression in muscle, and reduce glucose output from the liver through the inhibition of both glycogenolysis and gluconeogenesis, the combination of enhanced hepatic glucose release and the inhibition of muscle glucose uptake leads to relative or overt hyperglycaemia. This acute stress hyperglycaemia has been shown to be associated with increased rates of post-critical illness DM (168). Although it is likely that post-critical illness DM is a latent disease revealed by acute illness for many (169), evidence of increased rates of insulin resistance and metabolic syndrome that relate to the magnitude of traumatic injuries in UK veterans suggests this is not necessarily the case (170). Furthermore, hyperglycaemia and insulin resistance, which can continue alongside low grade inflammation for months beyond the acute phase of critical illness in the absence of overt DM, are known to contribute to sustained vascular dysfunction *via* endothelial glycocalyx disruption, the inhibition of endothelial nitric oxide mediated vasodilatation and aggressive pro-atherosclerotic myeloid behaviors (171–174). In this context the emergence of GDF-15 as a cardiovascular and all-cause mortality risk biomarker outside of ICU is notable.

Acute critical illness related weight loss involves a combination of adipose, muscle and skeletal compartment biomass depletion. Muscle mass is lost partly due to immobilization but in common with lipolysis, proteolysis also occurs due to the rewiring of systemic metabolism during critical illness, furnishing substrate particularly to the starved tricarboxylic acid cycle (175, 176). Brief reductions in cellular adenosine triphosphate (ATP) levels, which can occur due to many factors such as hypoxia, the activation of glycolysis and closely related β_2_-adrenergic activation of the Na^+^/K^+^-ATPase, result in a decrease in cellular energy charge which can be compensated for by the conversion of 2 molecules of adenosine diphosphate (ADP) to one molecule each of ATP and AMP. The generated adenosine monophosphate (AMP) in turn activates the cellular energy node 5′ AMP kinase (AMPK) which alters metabolic flux to generate more ATP by favoring catabolism over anabolism. Thus, the activation of fat and protein catabolism is intimately linked with the aforementioned alterations in lipid metabolism and insulin resistance, and is tied to glycolysis activation and the attempted restoration of cellular energy charge. Although glycolysis generates pyruvate which usually enters the tricarboxylic acid cycle (TCA), studies from critically ill patients show alterations in pyruvate dehydrogenase activity and defects in fatty acid beta-oxidation, associated with the development of lipid droplets in immune cells and multiple different non-adipose tissue beds (177–179). The biochemical bottleneck downstream of pyruvate and around long chain fatty acid metabolism coupled with the ability to utilize lactate to feed the TCA cycle is possibly why septic cardiac dysfunction is associated with an increased reliance on lactate as an energy substrate and a reduced use of FFA and ketones (180, 181).

Lipid droplets ordinarily harbor contact sites for mitochondria for the co-ordinate running of cellular metabolism, but in the presence of intracellular bacterial infection and presumably pathogen associated molecular pattern (PAMP) sensing, mitochondrial-lipid droplet contacts decrease, altering the flux of lipid metabolism while improving innate immunity through the secretion of antimicrobial lipoproteins (182). In keeping with their important role in immunity, lipid droplets also serve as platforms for nucleic acid detection, enhancing type 1 antiviral interferon responses and are also formed in response to endoplasmic reticulum stress and other forms of cellular stress (183). Therefore, these changes in cellular lipid content could be seen partly as an evolutionary conserved innate immune response to perceived pathogen threat and also a defensive sequestration of backed up cytotoxic lipids such as diacylglycerol. However, they come at a cost of an obligate deterioration in mitochondrial substrate processing when the number of contact sites decrease. Defects of mitochondrial fatty acid delivery and oxidation with subsequent lipid droplet accumulation that occur in the context of insulin resistance superficially appear similar to an accelerated from of metabolic syndrome associated fatty liver disease (MAFLD) and DM. However, acute phase metabolic and organ dysfunction abnormalities in critical illnesses appear to share greater overlap with those responsible for acute fatty liver of pregnancy, such as inherited defects in long chain 3-hydroxyacyl coenzyme A dehydrogenase (LCAD) and carnitine palmitoyl-transferase 1, both enzymes under the regulatory control of peroxisome proliferator-activated receptor (PPAR) pathways. Liver biopsy samples from acute fatty liver of pregnancy patients and from those with liver failure due to mitochondrial damaging therapeutics all converge histologically or biochemically on lipotoxicity and impairment of mitochondrial function at the level of beta oxidation, though the clinical context is clearly liver specific (184). In the case of non-liver critical illnesses such as sepsis, post-mortem histology reveals hepatic steatosis in roughly three quarters of patients (185). Furthermore, the same PPAR-α regulated defects in lipid handling and beta oxidation have been found in small cohorts of critically ill patients and a much larger cohort of stable cirrhotics with multi-organ failure (177–179, 186). This suggests that impaired beta oxidation occurs in the liver and also in a systemic manner in multiple organ failure and that the metabolic abnormalities that cause steatosis and an acute liver failure in AFLP merely signpost how the same process is likely to occur in other organs in critical illnesses (185, 187). In support of this abundant lipid droplets and impairments in fatty acid oxidation have also been found in the septic heart, acutely injured kidney and the skeletal muscle of critically ill patients, while reduced expression of PPAR-α has also been found in patients with poor sepsis outcomes (188–191). Findings of beta oxidation impairment associating with CKD stage, and lipid droplet accumulation in diabetic nephropathy highlight that these acute changes of metabolism in critical illness might lead to ongoing mitochondrial and chronic organ dysfunction in the context of residual inflammation and insulin resistance through lipotoxicity (192, 193). The importance of these changes and how they are also linked with acute changes in mitochondrial responses to stress and late immune and organ dysfunction are discussed further below.

### The Importance of the NLRP3/IL-1β/IL-6 Signaling Axis in Acute Illness and Cardiovascular Disease

Critical illness occurs when detected threat or injury, synergises with the dynamic host response to it, so that critical illness necessarily is associated with substantial activation of any of a number of pattern recognition receptors such as TLRs. The nucleotide-binding domain leucine-rich repeat and pyrin domain containing receptor 3 (NLRP3) inflammasome operates as a threat-detecting intracellular platform that is required for the formation of IL-1β from its precursor pro-IL-1β, and is instrumental in its cellular release through gasdermin membrane pores in response to detected DAMPs, PAMPs and harmful alterations in the cellular interior milieu, such as alterations in redox status and mitochondrial dysfunction (194). In keeping with this it has been suggested that familial Mediterranean fever, an autoinflammatory condition known to associate with cardiovascular disease and high IL-1β secretion through pyrin overactivity, may have emerged through selection pressures for enhanced resistance to epidemic infections such as Yersinia pestis (195, 196). Importantly, the induction of NLRP3 assembly and pro-IL-1β (and pro-IL-18) production is dependent on a priming signal which in modern times might originate from persistent low grade systemic inflammation as occurs in obesity or aging, or indeed following a first-hit acute inflammatory insult such as surgery/trauma or early infection. Secretion of the pro-inflammatory cytokine IL-1β (and its IL-1 superfamily member IL-18) is thus intricately linked to cellular stress and regulated, necrosis-like cell death (pyroptosis) which may be partly responsible for myocardial injuries that are seen post-surgically and in association with sepsis and other critical illnesses (197, 198). Although this implicates this pathway in pyroptosis, other forms of regulated cell-death (e.g., necroptosis) also implicated in cardiomyocyte death share a high degrees of cross-talk with it and may also be up or down-regulated (199). NLRP3 activation in cardiomyocytes has been shown in pre-clinical models to trigger spontaneous AF, and is upregulated in the atrial cardiomyocytes of patients with post-operative AF, implying that increased activation of this signaling pathway acutely and during convalescence may be an important contributing factor to paroxysmal AF and its consequences (120, 200).

IL-1β signals *via* its cytokine receptor IL-1R and induces the production of IL-6, which leads to C-reactive protein (CRP) production by the liver. In the aftermath of critical illness, residual inflammatory activity (measured by high sensitivity (hs)CRP) is very common and is associated with higher rates of metabolic syndrome and an elevated risk of death from cardiovascular causes (8, 73, 170). Other studies suggest both IL-1β and IL-6 levels remain elevated in over 30% of ICU survivors at 3-months while CRP is detectable in at least 60%, with correlation between functional outcomes and inflammatory state—this is partially in keeping with pre-clinical studies that suggest IL-1β activity is part of a disease tolerance programme that comes at the cost of fibrosis, cachexia and of course, cardiovascular disease risk (201, 202). As IL-1R mediates mtDNA release and a nucleic acid sensing (antiviral) response which should trigger cellular resilience through tolerance (203, 204), the involvement of persistent inflammation through IL-1β in chronicity may be a function of a dysregulated genetic tolerance programme that leads to a vicious cycle of chronic infection, inflammation and fibrosis (205). In addition to the primary findings of CANTOS where IL-1β repression resulted in a reduction in cardiovascular morbidity and mortality, residual inflammation after treatment with canakinumab (assessed through IL-6 and IL-18 tertiles) also associated with incident cardiovascular diseases (206). Furthermore, genetically determined decreases in IL-6 signaling reduce the likelihood of cardiovascular events in patients with IL-1β related cardiovascular risk factors, but not in those without (207). It therefore seems likely that IL-1β, IL-6 and possibly CRP activity after critical illness perpetuate vascular dysfunction and cardiovascular disease just as they do in the outpatient setting, and this is likely a consequence of ongoing processes linked to cell stress, inflammatory cell death and the engagement of a disease tolerance programme. Furthermore, pro-fibrotic AngII has also been shown to activate the NLRP3 inflammasome via AT_1_ receptor ligation, downstream NOX activation, with attendant vascular remodeling and renal inflammation (208, 209). Similarly, β_1_-adrenoceptor activation can also trigger myocardial NLRP3, contributing to immune cell ingress and myocardial fibrosis (210). In this regard it is notable that murine models of sepsis and acute inflammation show significant acceleration of the atherosclerotic process, with increased vascular macrophage infiltration seen in the recovery stage correlating with acute stage upregulation of inflammatory cytokines such as IL-6, and adhesion molecules in vasculature (42, 43). This finding in mice is further corroborated by epidemiological human data showing increased rates of cardiovascular pathology for many years following sepsis (23).

### Chronic Critical Illness Features a Senescent Adaptive Immune Phenotype Akin to That in Hypertension and Atherosclerosis

The recruitment of innate immune cells to the vascular and renal compartments is a phenomenon that is well-recognized in atherosclerosis, AKI and CKD (211, 212). However, many studies in humans and animals have also supported the concept of T-cell and macrophage involvement in the pathogenesis of hypertensive vascular dysfunction (213). CD4^+^ (T_h_1 & T_h_17), CD8^+^, and _γδ_T-cells have been shown to be important in driving hypertensive changes while regulatory T lymphocytes (T_reg_)and myeloid derived suppressor cells (MDSC) ameliorate hypertension related vascular dysfunction. Renal infiltrates of cytotoxic CD8^+^ T lymphocytes are also associated with vascular dysfunction and hypertension. Furthermore, it would appear that the CD8^+^ T-cells that are found in hypertensives have a greater degree of immunosenescence than age and sex-matched controls, and as such they produce greater quantities of Interferon (IFN)-γ, tumor necrosis factor (TNF)-β, granzyme B, and perforin (214). Studies of the immune landscape of symptomatic and asymptomatic atherosclerotic plaques also show large numbers of T-cells that are primed for granzyme and perforin secretion (212). These observations are important for understanding how cardiovascular and renal diseases might continue to progress in the immediate post-critical illness period. Immunosenescence is a characteristic of critical illness survivorship, and the abnormalities in the immune system with persistent inflammation take more than a year to return to normal levels (48, 215). T-cell Programmed Death (PD)-1 receptor positivity is a mark of exhaustion/anergy is both common in vulnerable atherosclerotic plaques and is a result of persistent inflammatory and hypoxic stimuli, such as that occurring during critical illness and infections (212, 216). Furthermore, findings that immunosenescence alone can drive tissue aging (217), and that biological age can be accurately predicted by both cardiovascular biomarker scores and an associated immune-aging score based on various markers of immunosenescence, such as T-cell PD-1 receptor positivity, lends further support to the hypothesis that the immunesenescence that follows critical illness results in a drive toward both cardiovascular disease and aging (218, 219). It is also notable that rates of viral reactivation in critical illness are high, though it is unclear whether re-activation of cytomegalovirus (CMV) and Epstein Barr Virus (EBV) exacerbate the T-cell immune dysfunction or are its epiphenomena (48, 220, 221). There is evidence that CMV infection is associated with hypertension and vascular dysfunction, and that viral persistence is associated with alterations in T-cell distributions and function similar to those found in persistent critical illness (222–225). Importantly, suppression of viral reactivation in critical care is safe and feasible, but so far has not shown significant mortality benefits, suggesting that latent processes driving functional immunosuppression are of greater importance than CMV disease in driving mortality and morbidity (226).

### Critical Illness Produces a Persistent and Trained Innate Immune Memory *via* Immunometabolic Changes Associated With Atherosclerotic Disease Progression

**Figure 1:**
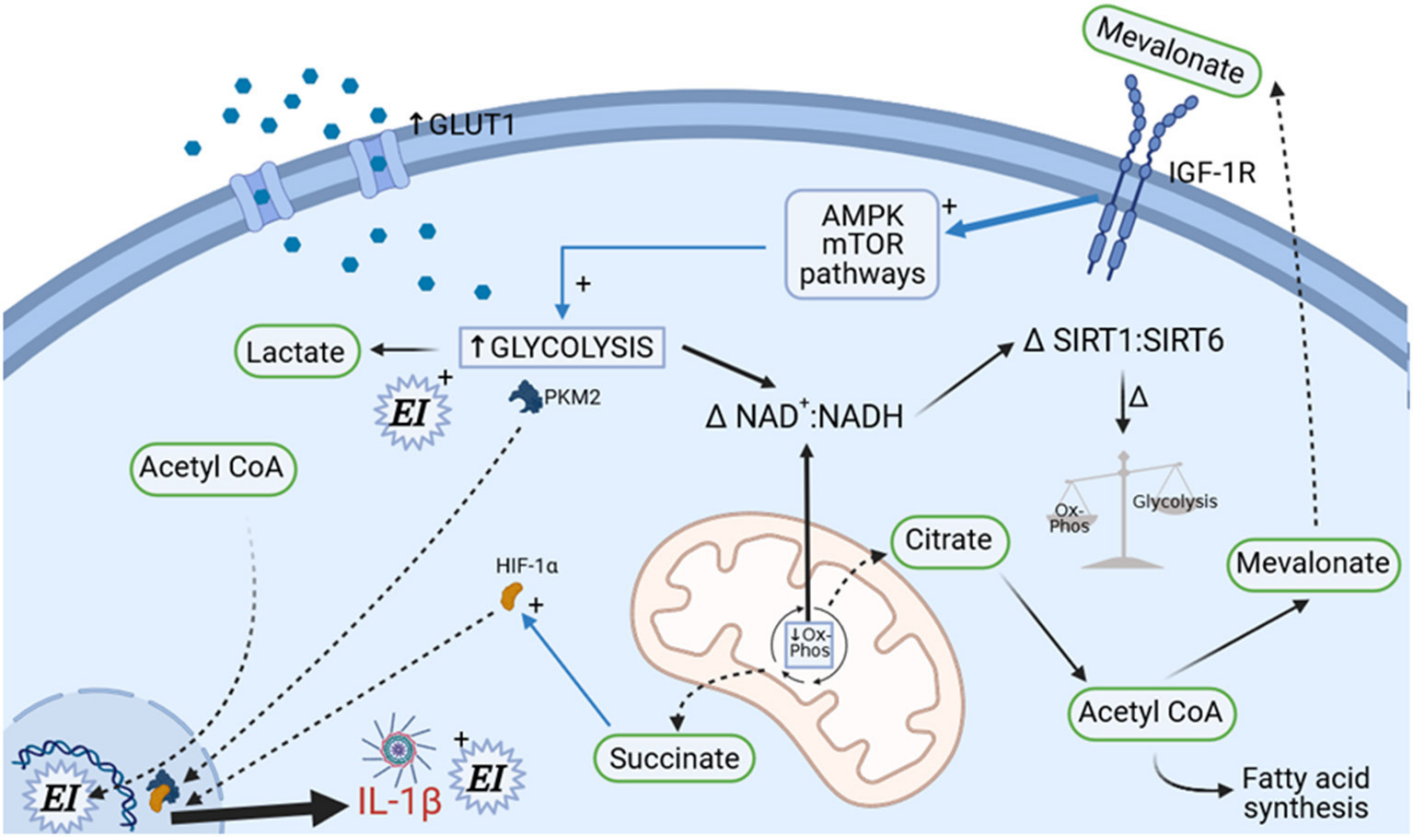
Immunometabolic determination of immune cell polarization. A complex set of metabolic changes occur under cell stress, involving upregulation of GLUT1 transporters, upregulation of cholesterol biosynthesis, decrease in mitochondrial oxidative phosphorylation (causing increased succinate formation), and upregulation of glycolytic enzyme activity. Cellular redox status (reflected in NAD^+^/NADH^+^ ratio) is consequently altered. These changes determine future immune cell behavior through alterations in epigenetic imprinting (EI), but also determine current immune cell cytokine responses *via* HIF-1α. The involvement of mitochondria (center in picture) is fundamental to this process; alterations in fusion/fission status under the control of mTOR, PGC1α, and Drp1 control immune cell functional state and might also directly contribute to organ dysfunction (not shown).

The traditional model of the innate immune system describes a system without any retained memory function. This concept has radically changed following the recognition that epigenetic reprogramming of myeloid precursors occurs after innate immune stimulation, resulting in a shift in innate immune responses to either trained or tolerant states (227). The proposed mechanisms for this reprogramming are sophisticated and relate immunometabolic changes that occur in infection with those that are seen in vascular dysfunction and atherosclerosis. These immunometabolic changes are intricately tied to the redox state of the cell (Figure 1).

The metabolic response to stress and infection involves altered cellular patterns of glycolysis and oxidative phosphorylation along with alterations in the NAD^+^/NADH balance of the cell. The glycaemic response to sepsis is well-recognized and has been clearly linked to upregulation of glycolytic pathways in immune cells with involvement of cellular energy sensors such as mechanistic target of rapamycin (mTOR) and AMPK (228, 229). Enhanced glycolysis triggers alterations in the TCA cycle resulting in mitochondrial succinate generation and also driving nuclear translocation of the glycolytic enzyme pyruvate kinase M2 (PKM2). Nuclear PKM2 dimerises and forms a complex with the transcription factor hypoxia inducible factor (HIF)-1α (which is activated by succinate), resulting in acceleration of the Warburg effect, greater lactate production, and pro-inflammatory cytokine generation in macrophages. This HIF-1α related phenomenon occurs in both the atherosclerotic and septic settings and may also mediate enhanced neutrophil mobilization from the bone marrow through lactate (230–235). These metabolic changes are associated with increased intracellular concentrations of mevalonate, a product of the cholesterol biosynthesis pathway. Mevalonate signals in an autocrine and paracrine fashion *via* insulin-like growth factor-1 (IGF-1) which further enhances glycolysis *via* sensitization of mTOR and AMPK. Crucially, mevalonate also translocates to the cell nucleus where it is responsible for epigenetic modification of the monocyte and macrophage genome such that host defense molecules and cytokine response (particularly IL-1β) is more vigorous on subsequent exposure to stressors. Statins inhibit mevalonate production and in addition to their effect on circulating cholesterol also stabilize atherosclerotic plaques through a reduction in monocyte and plaque macrophage trained immunity and hyperinflammation (234, 236). Whether their effects on monocyte and macrophage immunometabolism is an important mechanism in their potential action in hyperinflammatory ARDS is unclear at this time (237).

The macrophage phenotype of primed glycolysis (as opposed to mitochondrial dependent oxidative-phosphorylation) coupled with appropriate histone modifications on the macrophage genome and a tendency to IL-1β production has been found in the cells of vulnerable atherosclerotic plaques in several studies—the so-called M1 phenotype (238). Furthermore, in addition to LPS/TLR4 and β-d-Glucan/Dectin-1 signaling (sepsis activated pathways), oxidized LDL, aldosterone and catecholamines have also been shown to trigger trained immunity (227, 239, 240). In order for trained immunity to persist, it must be imprinted in the bone marrow niche. This training process occurs in haematopoietic progenitor cells causing myeloid precursors to be released into the circulation primed in this state (241). In summary, an epigenetic training of monocytes, macrophages and T-cells (see below) which is associated with atherosclerosis and vascular dysfunction follows on from the altered redox status and metabolite profile that itself results from the cellular stress of critical illness and infection (242).

### Trained Immunity Is Intrinsically Linked to Mitochondrial and Metabolic Changes That May Also Explain Chronic Organ Dysfunction

When Ox-phos is interrupted, such as in ischaemia, the TCA intermediate succinate accumulates (243). In stressed cardiomyocytes succinate signals *via* its receptor GPR91, leading to mitochondrial fission and dysfunction, and cardiomyocyte apoptosis (244). This has been shown to be an important mechanism in myocardial post-ischaemia-reperfusion dysfunction, but whether this also is true for septic cardiac dysfunction remains unknown. Mitochondrial fission involving succinate, Dynamin related protein-1 (Drp1) and the mTOR regulated transcription factor PPAR-γ co-activator 1 (PGC1α), is part of a pattern of metabolic change occurring under cell stress that is required to drive the switch from resting state to the pro-inflammatory phenotype, thus linking altered mitochondrial metabolism to cell stress/death and pro-inflammatory IL-1β secretion (245–248). On the other hand, fused mitochondria show more efficient oxidative phosphorylation and associate with the endoplasmic reticulum (notably, the site of lipid droplet formation) where they sequester calcium. This is associated with the alternately activated (M2) macrophage phenotype and Th_2_ responses (249–251).

However, the epigenetic imprinting of T-cells relates to their anergic/exhausted status too, so that epigenetic reprogramming of immune cells as previously described is not solely an innate immune process, nor one which always enhances an inflammatory immune response (252). Indeed the late phase of acute illness which is characterized by T-cell anergy and PD-1 upregulation is also accompanied by a switch of leukocytes back from a glycolytic pro-inflammatory state to an immunotolerant mode where ox-phos is also inhibited (this is as opposed to an ordered return to ox-phos with preserved glycolysis) (253, 254). This may provide a metabolic explanation for the increased propensity to infections seen in late stage critical illness—a broad dysfunction of immune cell metabolism means cells are sluggish in responding to a secondary or residual stimulus due to a switch at the cellular level. This presumably costs the host less energy than continual crudescence, but possibly represents a high risk strategy of immune tolerance that may also result in reduced pathogen clearance (255). This is somewhat in keeping with ideas regarding the two strategies by which organisms handle infection, resistance and tolerance. Resistance refers to host strategies that ensure survival by actively reducing pathogen burden, whereas tolerance programs improve host survival at any given level of pathogen load through resilience. Although complimentary, these strategies may dynamically change in dominance depending on the stage of illness, the switch of strategy being determined by energetic considerations that have been selected through evolution (256, 257). Studies show that reductions in complex 1 activity of the electron transport chain in mitochondria, whether through hormetic processes or ribosomally targeted drugs (258, 259), results in changes in mitochondrial substrate handling that increase disease tolerance. In the case of exposure to LPS, ox-phos and fatty oxidation decrease with subsequent reductions in pro-inflammatory epigenetic imprinting through histone acetylation and a concurrent switch from pro-inflammatory M1 to tolerant M2 macrophages. These metabolic changes are in keeping with a reduction in mitochondrial oxygen consumption that is under the control of aforementioned mTOR and PGC-1α (247).

This immunoparesis would tend to decrease the risk of autoimmunity to neoantigens following severe acute inflammation—the evidence that autoimmunity can occur in response to anti-PD-1 therapy in cancer, supports this response representing a deleterious but programmed protective mechanism (255, 260). The importance of epigenetic mechanisms linked to cell metabolism is further demonstrated by the fact that this bi-directional, NAD^+^ dependent switch of ox-phos to glycolysis, and the switch to T-cell anergy, is mediated by histone modifying sirtuin enzymes at the epigenetic level (261, 262). It is then tempting to speculate that in those critically ill patients with the worst outcomes (death or chronicity) an early pro-inflammatory stage could result in decreases in cellular NAD^+^ and a subsequent failure of SIRT3 to prevent LCAD and glycolytic enzyme hyperacetylation—this would not only result in impaired fatty acid oxidation and subsequent cellular lipid accumulation, but also the acetylation of numerous mitochondrial proteins and glycolytic enzymes with subsequent immunoparesis and chronic organ dysfunction, as seen in sepsis and CKD (177, 190, 193, 253, 263).

### Critical Illness Produces Bone Marrow Stress, and May Produce Somatic Mutations in the Haematopoietic Stem Cell Niche Favoring Myeloid Skewing, Clonality, and NLRP3 Inflammasome-Dependent Cardiovascular Disease

Critical illness presents multiple stresses on the bone marrow. Immobility, inactivity and sedentarism are associated with prolonged increases in bone marrow fat content, which leads to increased leptin signaling and inflammatory monocytic output (264, 265). Furthermore, there is sustained cytokine, glucocorticoid and sympathetic nervous system activity that places additional replicative pressure on this niche with a resultant myeloid skewing to its output (266, 267). Dysglycaemia, which commonly accompanies the insulin resistance of severe illness and continues in convalescence, also leads to increased myelopoiesis with inflammatory monocytosis and neutrophilia, at least one mechanism being *via* S100A8/A9-mediated activation of the receptor for advanced glycation end-products (174). Sleep, which normally places a brake on bone marrow replication rates, is severely disrupted during severe critical illnesses and remains disrupted for a long time after and this will further compound bone marrow replicative pressure in convalescence (268, 269). The combined effect of these disruptions during critical illness convalescence would be to result in a sustained increase in myeloid progenitor replication rates, a myeloid skewed output with an inflammatory monocyte and neutrophil cohort, and the propagation of atherosclerotic disease and myocardial injuries.

The burden of somatic mutations in the human body is greater than previously appreciated (270). Physico-chemical attack on the genome, for example by ultraviolet radiation and cigarette smoking, results in DNA damage and consequent repair mechanisms which are imperfect, thus resulting in mutation. This also occurs under other circumstances where free-radical mediated damage is increased, such as under conditions of severe inflammation as seen in critical care (271, 272). Emergency myelopoiesis is a feature of the acute response to inflammation and is dependent on epigenetic modifying enzymes such as Tet2 (273). However, inflammation favors not only myelopoiesis and increased rates of somatic mutation, but also increases the probability of myeloid clonal survival (274). A specific form of myeloid clonal survival with a blood variant fraction of 2% or more [termed clonal haematopoiesis (CH) of indeterminate potential (CHIP)] is very strongly associated with driver mutations in genes for epigenetic regulators such as TET2, DNMT3A, ASXL1, and also in genes that code for signaling molecules such as JAK2 and TP53 (275). CHIP is also associated with age, being highly prevalent above the age of 60 years, coupled with an increased incidence of cardiovascular disease (276). These clonal changes are of indeterminate potential as they are preleukaemic, though only a small proportion of those with CHIP go on to develop myelodysplastic syndromes and acute myeloid leukaemias. These preleukaemic changes are driven by TLR2-sensed microbial signals as may be found in critically ill patients with infections or with gut barrier dysfunction (277). Studies support a bi-directionality of causation between inflammation (vascular or otherwise) and CHIP, and as CH exacerbates insulin resistance in preclinical models (278, 279), the hypothesis that critical illnesses can drive CHIP must be taken seriously (280).

Both atherosclerosis and heart failure are mechanistically linked with clonality, Tet2 and Dnmt3a loss of function, consequent increased activation of the NLRP3 inflammasome and IL-1β and IL-6 production in monocytes (281–283). IL-1β antagonism, which mechanistically should reduce IL-6 and hsCRP, reduced cardiovascular event rates only in those with therapy sensitive decreases in hsCRP in the CANTOS trial (44). Considering that Tet2 is required for IL-6 driven inflammation repression whereas Dnmt3a represses mast cell inflammatory responses (284, 285), if treatment responders in CANTOS had a high incidence of CHIP while the treatment non-responders didn't, a mechanistic pathway with supportive data exits to explain this observation (286). This latter paradigm where CHIP drives post-acute illness adverse outcomes is supported by survival data from patients undergoing trans-catheter aortic valve implantation (287). The finding that CHIP is a cardiovascular risk factor, increases the propensity to certain types of infection, and that Tet2 dysfunction leads to sub-optimal emergency myelopoiesis (273), means investigating whether CHIP has a role in NLRP3 hyperactivation, peri-operative and ICU-acquired non-ischaemic myocardial injuries, the inability to resolve inflammation is a high priority particularly as it may be important targetable driver of hyperinflammation in both the acute and survivor phases of critical illnesses (288).

### Deleterious Mineralocorticoid Signaling Activates the Immune System and Occurs in Critical Illness

Studies in primary hyperaldosteronism show that mineralocorticoid receptor (MR) agonism without an acute pathological insult is deleterious to the cardiovascular and renal systems (289). Renal, cardiac and vascular T-cell infiltration, IFN-γ production and deleterious remodeling in the context of hypertension has been shown to be modifiable by action at T-cell mineralocorticoid receptors (MR), supporting the role of immune infiltrates in cardiovascular and renal chronic disease progression (290, 291). Similarly MRs are present in myeloid cells and are involved in polarization to a pro-inflammatory phenotype with receptor blockade by antagonists reducing cardiac hypertrophy, fibrosis, and vascular damage following pathological insults (292). Fibrotic remodeling in the context of MR agonism can occur without clinical hypertension, suggesting that vascular dysfunction and tissue remodeling is a feature of a specific immune response which often predates hypertensive change (293). This suggests that a disease process producing similar immune changes, such as critical illness, could also begin the cascade of tissue remodeling resulting in these disease phenotypes without overt signs of hypertension. It is also noteworthy that adverse tissue remodeling through MR activation depends on immune cell and adipose released neutrophil gelatinase-associated lipocalin (NGAL) (294, 295). NGAL is substantially upregulated during critical illness and has also been found at elevated levels in the bloodstream of patients with heart disease (independent of renal function) where it carries prognostic significance (294, 296–298). In addition to the genomic MR pathway, there are additional non-genomic MR pathways that are related to adverse tissue remodeling—namely the transactivation of epithelial growth factor receptors (EGFR), the IGF-1 receptor pathway, and the AT_1_ receptor/NOX pathway (299). NOX, AT_1_, and IGF-1 have already been described as carrying key roles in the proposed pathophysiology above and are heavily involved in immunometabolic change and epigenetic training.

The MR also has an important role in the regulation of the cardiovascular system at the level of the paraventricular nucleus (PVN) of the hypothalamus. Hypothalamic AT_1_-receptor activation and endotoxin-mediated TLR activation, as occurs in sepsis leads to increased hypothalamic NOX and NFκB activity, which in turn has been shown to deleteriously affect autonomic tone and cardiac remodeling (300). It also appears that circulating pro-inflammatory cytokines have the same effect on the PVN and this is mediated by perivascular macrophages (301). These changes in hypothalamic NOX, nuclear factor kappa B (NFκB), the associated sympathetic overactivity and dysautonomia are seen in aging, in sedentarism, in hypertensives, after myocardial infarction and in heart failure, and it is particularly notable that microglia (central nervous system macrophages), play a prominent role in this (302–305). Furthermore, continual centrally mediated sympathetic activation has been found in pre-hypertensives where it contributes to deleterious cardiovascular remodeling, and patients with dysautonomia have been shown to have cardiac hypertrophy which is mediated by MR mediated supine hypertension despite normal daytime blood pressure and aldosterone levels (306, 307). Activation of hypothalamic MRs enhances the AT_1_-mediated effect on NOX while antagonism of hypothalamic MRs is associated with improvements in dysautonomia, reduced hypothalamic NOX activation and reduced hypertension (308–310). This central mechanism of dysautonomia, partially driven by oxidative stress is likely an important one for the recovering critically ill patient who often has encountered a degree of autonomic dysfunction (311). Further evidence that this may be of significance is that significantly increased free radical mediated apoptotic change has been found in the microglial fraction of hypothalamuses of septic shock patients (312).

The acute response to severe illness involves activation of the sympathetic and renin-angiotensin-aldosterone systems. Different studies suggest aldosterone levels are either higher or lower than normal in severe disease (313, 314). However, pathological MR agonism is not solely an aldosterone related phenomenon. Cortisol and aldosterone both have the same affinity for and can signal *via* the MR. Furthermore, functional plasma levels of cortisol are 100-fold higher than those of aldosterone. Intracellular enzyme systems (11-β hydroxysteroid dehydrogenases I & II) and cellular redox status ensure aldosterone selectivity for the MR in health (315). During physiological stress increased circulating cortisol levels induce these enzymes so that intracellular cortisol levels increase further, whereas an alteration of the cellular redox status affects the mechanisms of transcriptional repression that normally inhibit cortisol-dependent pathways of MR activation (316, 317). Therefore under stress, peripheral and central MRs can become activated by glucocorticoids, as suggested by experimental studies (308, 318). The beneficial effects of MR antagonists in patients with chronic cardiovascular pathology where plasma aldosterone levels are normal is suspected to be mediated in this way (319). Notwithstanding that, the picture during critical illness is uncertain as dynamic changes in plasma cortisol and aldosterone during this time are well-recognized though poorly understood, and furthermore tissue resistance to steroid hormones in sepsis is strongly suspected to exist (320). Nevertheless, it is clear that the MR is a receptor with immunomodulatory actions and one which modifies both redox status (*via* NOX & AT_1_) and is involved in tissue remodeling (*via* EGFR/IGF-1 pathways). This and the known adverse cardiorenal outcomes following severe critical illnesses is the basis for the anticipated Dapa-SSCILL randomized controlled trial (12).

### The Potential Role of the Clinicians' Fluid Therapies and Interstitial Sodium

During periods of shock, large volumes of intravenous resuscitation fluids are used by clinicians to restore the patient's intravascular volume. Although some degree of fluid resuscitation is almost invariably necessary, the optimal level for any individual patient is not defined. Furthermore, the use of resuscitative fluid therapy leads to atrial stretch and natriuretic peptide induced endothelial dysfunction, which may contribute to further acute organ dysfunction, and persists to some degree during convalescence (321, 322). TLR4 agonists such as endotoxin and resuscitation fluid volume have also been shown to induce (or associate with) the transformation of mature and progenitor endothelial cells to a fibroblast-like form, in keeping with the process of endothelial-to-mesenchymal transition (EMT) (323, 324). This important process which also involves TGF-β signaling is suspected to be centrally involved in multiple cardiovascular pathologies including atherosclerosis, leading weight to the idea that acute severe disease transitions to chronic (325).

Following resuscitation there is often a prolonged period where intravenous fluids continue to be delivered for either maintenance or as part of drug delivery. This is an enormous sodium burden for the critically ill patient, which is not easily mobilized (326, 327). Although plasma levels of sodium vary in critically ill patients, it is unclear whether vascular interstitial sodium concentrations are in the same range as plasma, or like hypertensive patients are potentially much higher. This has not been widely studied in the critically ill population but if interstitial sodium is and remains elevated as suggested by one small study in patients with AKI, then this elevation could also trigger antigen presenting dendritic and effector T_h_17 cells to exacerbate deleterious immune recruitment to the vasculature as in hypertensive patients (213, 328, 329).

**Figure 2:**
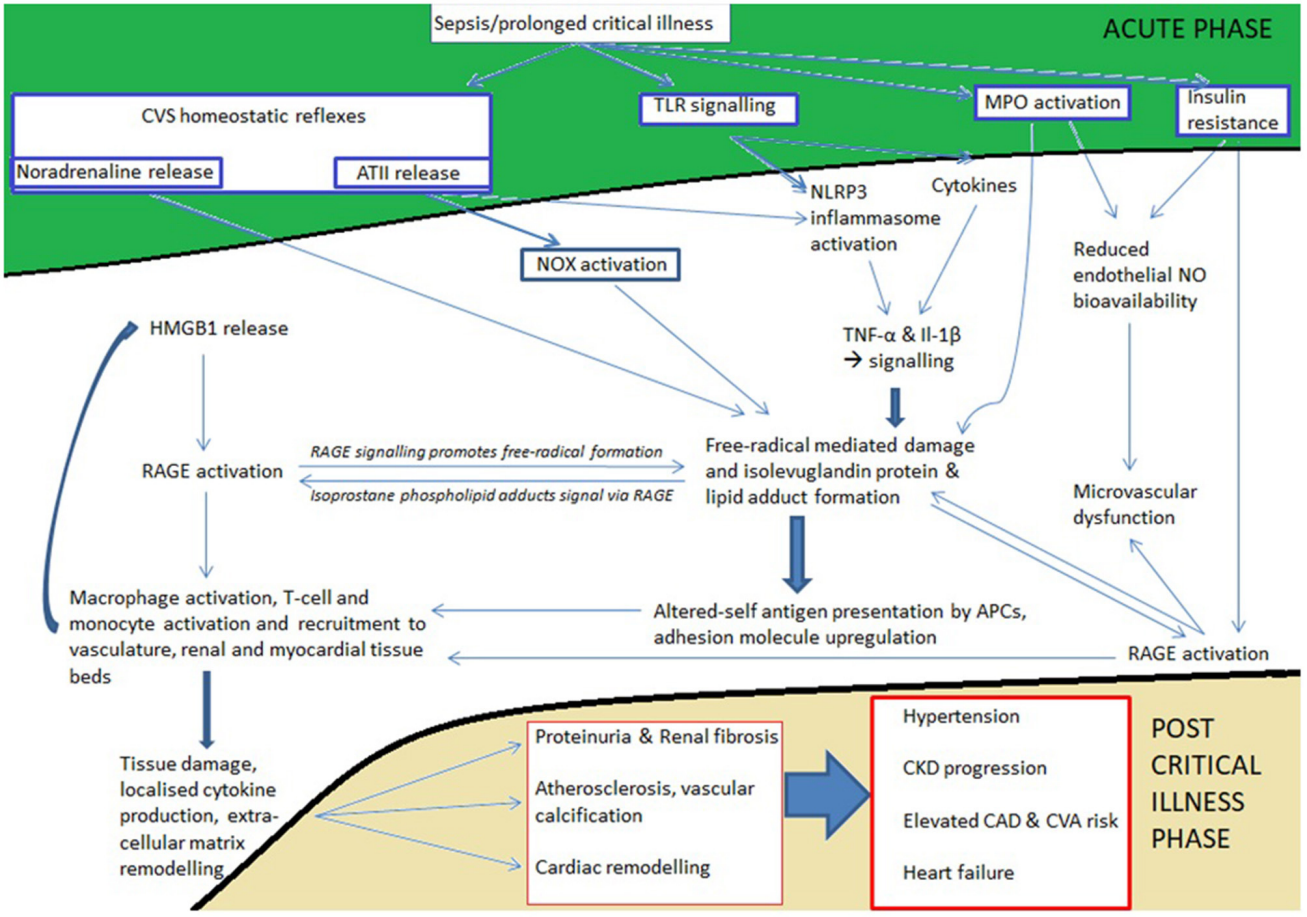
Outline of how acute inflammation leads to chronicity affecting the cardiovascular and renal systems. Areas in green are triggered during the onset of acute inflammation whereas areas in gold become apparent during survivorship. The white area between acute and chronic phases represents the cellular processes precipitated (*de novo* disease) or accelerated (disease already exists) by acute inflammation; these processes continue during survivorship because antigen presentation and deleterious immune infiltrates have become established. Note that none of the mechanisms in the white area are reliant on traditional risk factors of hypertension, dyslipidaemia, obesity, and diabetes for triggering the chronic diseases in gold. Nevertheless, the recently elucidated inflammatory and immune mechanisms underlying vascular dysfunction, hypertension, and atherosclerosis are central to triggering these chronic diseases. What is not specifically highlighted is the legacy effect of epigenetic reprogramming (trained immunity) due to the effects of altered cellular redox status and accelerated glycolysis on macrophages, monocytes, and T-cells.

Taking all this evidence together suggests that over the course of critical illness, severe acute inflammation (ROS generation, altered self-antigen presentation, high circulating levels of IL-1β, IFN-γ, and other pro-inflammatory cytokines), Ang II, catecholamines, and potentially the sodium load of fluid resuscitation synergise and produce an adverse cardiovascular and renal immune infiltrate. This creates (or accelerates pre-existing) cardiovascular and renal disease by virtue of an immunometabolic driven, long-lived epigenetic reprogramming of the immune system which in turn drives chronic low grade inflammation beyond the phase of critical illness. This chronic inflammation continues to drive pathology just as it does in patients with chronic diseases who have never had a critical illness but who go on to develop T2DM, CKD and cardiovascular diseases. The aforementioned mechanisms would imply a dose response relating to the severity of inflammation and likelihood of consequent cardiovascular and renal disease—indeed this is seen. Furthermore, they provide explanations for chronic illness pathologies in critically ill patients despite the absence of traditional risk factors, and despite significant periods of time where blood pressure is not elevated (Figure 2).

## Parallels in Critical COVID-19 Disease

The COVID-19 pandemic resulted in a global surge of often lengthy ICU admissions, predominantly for respiratory failure and ARDS. In common with ARDS, sepsis and other severe organ dysfunction syndrome patients, many critical-COVID-19 survivors have been left with chronic problems that continue to impede function at 2-year follow-up (330). Although critical-COVID-19 survivors frequently describe new problems, predominatly related to dyspnoea and limited functional capacity, some of these problems arise from SARS-CoV-2 viral infection with no, or only mild-moderate acute symptoms and therefore may not be related to critical illness *per se* (331). Nonetheless, studies clearly show that critical COVID-19 disease is associated with a significantly higher likelihood of a range of post-infection CVS diseases in addition to cognitive and functional impairments when compared to less severe disease, even in patients who have never experienced CVS disease before (26, 330). Although some of these functional problems may relate to the degree of slow-resolving pulmonary damage that was acutely sustained (332), this does suggest that critical illness and its associated biology induce maladaptive pathways of disease resolution in COVID-19 convalescence that go beyond those that are specific to long-COVID. Such common pathways might include those highlighted earlier which are involved in AKI to CKD transition (333, 334). Similarly, though SARS-CoV-2 infects cells by entry through the ACE receptor (335), direct pathogen-mediated CVS disease and inflammatory cell death with continued endotheliopathy (possibly in the face of a latent, active, viral reservoir) appears a convergent mode of post-ICU CVS dysfunction that may be especially pronounced after SARS-CoV-2 infection (336–338). Critical COVID-19 disease is also an important determinant of subjective long-term non-recovery, and residual inflammation (CRP) and coagulopathy are associated with worse subjective functional and cognitive outcomes too (332, 339). Importantly, the link between interferon-mediated (antiviral) inflammation, neurodegeneration and associated cognitive decline is increasingly appreciated but may also be a consequence of COVID-19 other inflammatory insults seen in ICU (e.g., trauma) that share similarity with nucleic acid activated stress pathways (340–342). These interferon-driven processes appear to be persistently active due to ongoing immune activation in those with long-COVID but it is unclear whether a similar persistence of interferon signaling occurs in convalescence after other critical illnesses (343). However, as in other critical illnesses, T-cell exhaustion is more likely found in severe acute COVID-19 disease (344), continues in convalescence, and is associated with mitochondrial and cellular metabolic dysfunction (345). It is therefore likely that further COVID-19 disease, long-COVID and post-critical COVID-19 recovery research will continue to highlight new and shared pathways of post-ICU CVS and other chronicities.

## Conclusion

The high levels of inflammation and reactive oxygen species (ROS) seen in severe critical illness generate an immune-mediated onset of *de novo*, or acceleration of pre-existing, cardiovascular disease. The CVS and renal disease that is seen therefore does not necessarily relate to traditional risk factors which tend to be normal or depressed acutely (such as blood pressure, lipid profile, HbA1c) at the onset of critical illness survival. The presence of continued low grade inflammation and immune changes that are slow to resolve following critical illness create an environment where adverse tissue remodeling continues, endothelial dysfunction is sustained, metabolic syndrome can take hold, and the risk of CVS and renal disease is elevated. In order to arrest disease progression an anti-inflammatory therapy is mandated which is sensitive to the fragile and immunosuppressed state of the critically ill.

## Author Contributions

All authors contributed equally to writing, proof reading, and editing. All authors contributed to the article and approved the submitted version.

## Conflict of Interest

The authors declare that the research was conducted in the absence of any commercial or financial relationships that could be construed as a potential conflict of interest.

## Publisher's Note

All claims expressed in this article are solely those of the authors and do not necessarily represent those of their affiliated organizations, or those of the publisher, the editors and the reviewers. Any product that may be evaluated in this article, or claim that may be made by its manufacturer, is not guaranteed or endorsed by the publisher.
